# Champagne Groove Lipectomy: A Safe Technique to Contour the Upper Abdomen in Abdominoplasty

**Published:** 2017-03-06

**Authors:** Ron Brooks, Jonathan Nguyen, Saeed Chowdhry, John Paul Tutela, Sean Kelishadi, David Yonick, Joshua Choo, Bradon J. Wilhelmi

**Affiliations:** University of Louisville, Louisville, Kentucky

**Keywords:** champagne groove lipectomy, upper abdomen contouring, abdominoplasty techniques, lipoabdominoplasty alternative, supra-scarpal fat excision

## Abstract

**Objective:** Combined liposuction and abdominoplasty, or lipoabdominoplasty, is particularly helpful in sculpting a more aesthetically pleasing abdominal contour, particularly in the supraumbilical midline groove. This groove, coined the “champagne groove” by one of our patients, is a frequently sought-after attribute by patients. However, liposuction adds time and cost to an already costly abdominoplasty. We sought to create this groove without the addition of liposuction, utilizing what we call a champagne groove lipectomy. This study reports on our champagne groove lipectomy technique and compares our complication rates with those reported in the literature for standard abdominoplasty techniques. **Methods:** This is a retrospective review of a single surgeon's experience at our institution over a 6-year period (2007-2012). A total of 74 patients undergoing consecutive abdominoplasty were studied, all female nonsmokers. Two groups were recognized: 64 of 74 patients underwent abdominoplasty, partial belt lipectomy, and champagne groove lipectomy, while 10 of 74 patients underwent fleur-de-lis abdominoplasty without champagne groove lipectomy. **Results:** Overall, 10 of 74 patients (13.5%) suffered some type of complication, which compares favorably with reported rates in the literature. The majority of complications were related to delayed wound healing or superficial wound dehiscence. Among those patients who underwent champagne groove lipectomy, complications occurred in 6 of 64 patients (9.3%), versus 4 of 10 (40%) patients undergoing fleur-de-lis abdominoplasty. **Conclusions:** Champagne groove lipectomy is a cost-effective alternative to lipoabdominoplasty for achieving an aesthetically pleasing upper midline abdominal contour, with complication rates comparing favorably with those reported in the literature.

Abdominoplasty (AP) continues to be one of the most common cosmetic surgical procedures done in the United States, being the fifth most common procedure performed in 2015, according to ASPS statistics^1^. There have been numerous AP techniques and modifications that have been described since Kelly's original description in 1899.^2^^,^^3^ Regardless of the technique employed, maintenance of adequate blood supply to the abdominal wall is critical to avoid local wound complications. Huger^4^ described 3 vascular zones of the abdominal wall, and, more recently, Matarasso^5^^,^^6^ described areas of the abdominal wall that can safely undergo liposuction during AP versus those that should be approached with caution.

Combined liposuction and AP, or lipoabdominoplasty, was once considered controversial due to the concern for increased disruption of the vascular supply to the abdominal wall with resulting skin necrosis, although many studies have now shown this to be safe with a similar complication profile to standard AP.^7^^-^9 Lipoabdominoplasty is particularly helpful in sculpting a more aesthetically pleasing abdominal contour, particularly in the supraumbilical midline groove. This groove, coined the “champagne groove”, is a postoperative attribute frequently sought by many of our patients. Despite the ability of liposuction to easily create the champagne groove, it certainly adds time and cost to an already costly procedure for many patients, which is a particular concern in our patient population. In addition, some literature suggests that combined liposuction with AP may increase seroma rates.^10^ We thus sought to create this groove without the addition of liposuction, utilizing what we call a champagne groove lipectomy (CGL). This study reports on our CGL technique and compares our complication rates with those reported in the literature.

## METHODS

This is a retrospective review of a single surgeon's experience at our institution over a 6-year period (2007-2012). All patients undergoing any type of AP technique were included, resulting in 74 consecutive patients to be studied. All patients were nonsmokers and all were women, ages ranging from 24 to 67 years with an average age of 43 years. Two groups were recognized: 64 of 74 patients underwent AP, partial belt lipectomy (PBL), and CGL, while 10 of 74 patients underwent fleur-de-lis (FDL) AP without CGL.

### Technique

**Figure 1-2:**
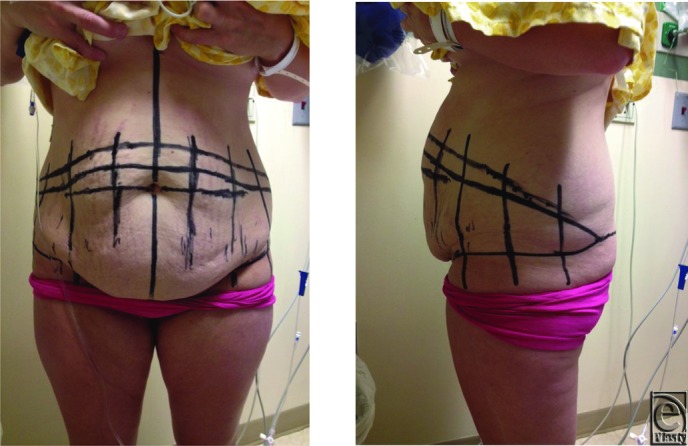
Preoperative markings for the abdominoplasty, partial belt, and champagne groove.

**Figure 3-5:**
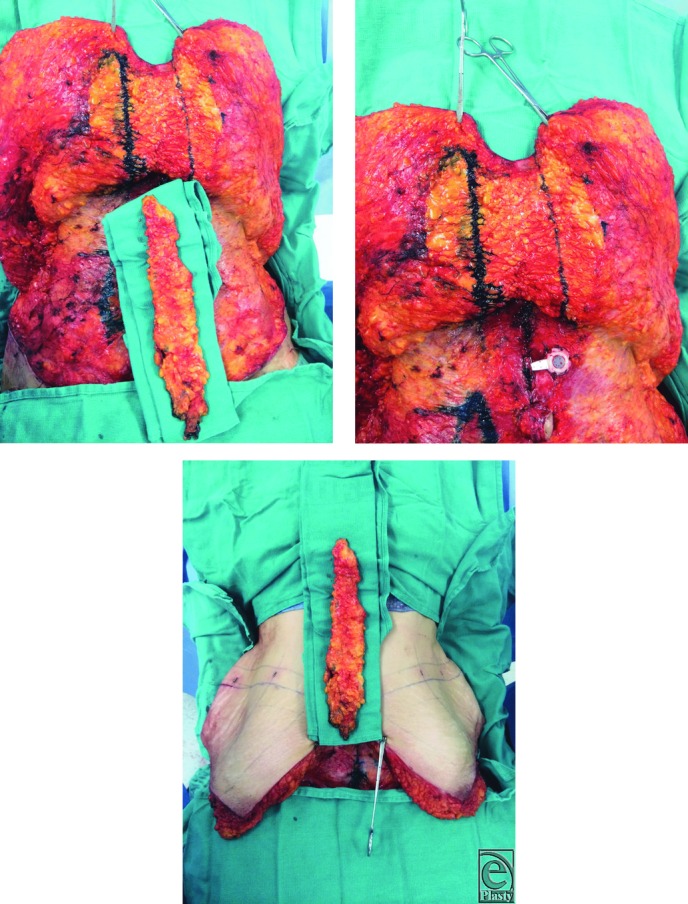
Creation of champagne groove—excision of 2- to 6-cm suprascarpal fat on either side of the midline.

**Figure 6:**
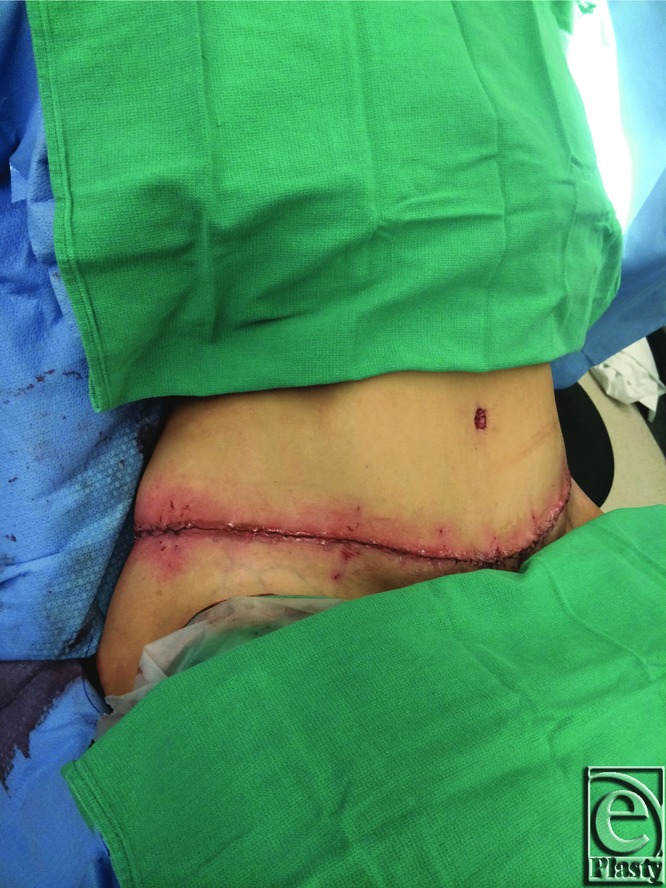
Wound closure.

For patients undergoing AP/PBL/CGL, a standard AP incision along the inferior border of the skin flap to be removed was performed and carried out laterally onto the flanks ending near the posterior axillary line (see Figs 1 and 2). Our senior surgeon typically performs a PBL as opposed to standard AP to correct the dog-ear deformity that often develops in these patients postoperatively when only an AP is performed. In his experience, revision of a dog-ear deformity was not uncommon with standard AP; thus, performing the PBL would ideally prevent the need for revisional surgery in the future. Superior undermining of the skin flap was then performed, with dissection above the umbilicus to the xiphoid typically limited to a 6- to 8-cm strip centrally. Lateral undermining was performed as needed to allow for adequate redraping, which is often near the costal margins. Fascial plication was then performed in the midline. If there continued to be laxity laterally after midline plication, lateral plication was also performed. Creating the champagne groove was done next by excising a superficial layer of fat anterior to Scarpa's fascia 2 to 4 cm on either side of the midline from the umbilicus to the xiphoid (see Figs 3-5). Umbilical transposition was performed on all patients prior to wound closure. The wounds were then closed in a layered fashion and 2 drains were typically placed on either side (see Fig 6).

For patients undergoing an FDL-type excision, a standard AP approach was used but undermining is typically not as aggressive as mentioned earlier, such that the undermining is not more than the amount of skin to be excised. Fascial plication is performed as mentioned earlier, and skin excision is then performed in both horizontal and vertical dimensions. Wounds were closed as described earlier.

## RESULTS

**Figure 7:**
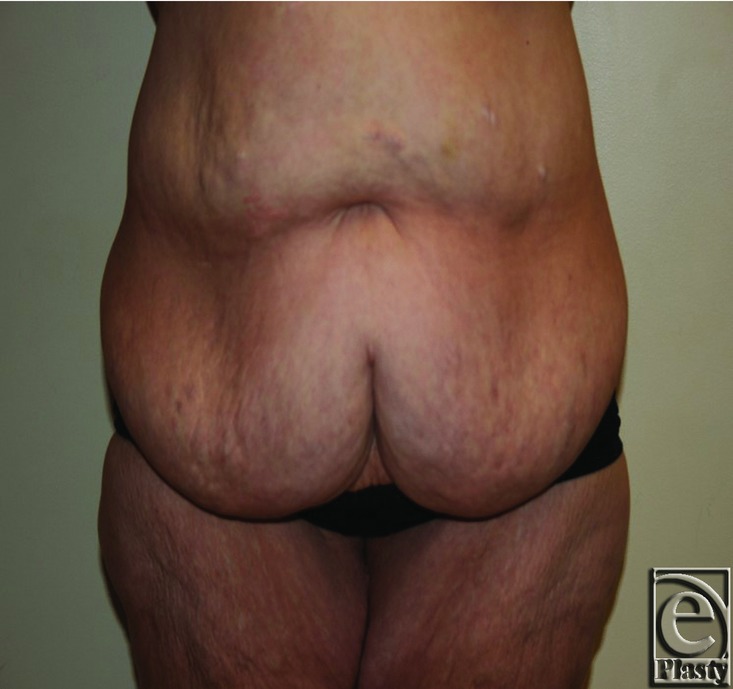
Preoperative photograph.

**Figure 8:**
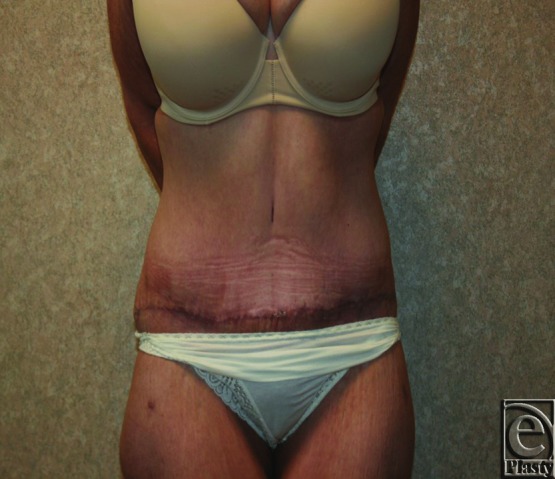
Three-month postoperative photograph.

The champagne groove technique resulted in aesthetically pleasing outcomes (see Figs 7 and 8). Overall, 10 of 74 patients (13.5%) suffered some type of complication, which compares favorably with reported rates in the literature.^11^^,^^12^ The majority of these complications (70%) were related to delayed wound healing or superficial wound dehiscence. Other complications included seroma, superficial wound infection requiring oral antibiotic therapy, and contour irregularity requiring revision at 1 year. Among those patients who underwent AP/PBL/CGL, complications occurred in 6 of 64 patients (9.3%), versus 4 of 10 patients undergoing FDL AP. At 3-month follow-up, the patients were pleased with their outcome.

## DISCUSSION/CONCLUSION

Given that AP continues to be one of the most commonly performed plastic surgery procedures done yearly, having a full armamentarium of techniques for each individual patient is beneficial. We are certainly not suggesting that our champagne groove technique replace liposuction, but it does provide a comparable, efficient alternative for midline contouring, with a similar risk profile. Certainly, maintenance of adequate blood flow to the abdominal flaps is crucial to any successful AP, and our CGL did not show any increased risk of skin necrosis. However, we also do not perform this suprascarpal fat excision in patients who are smokers. Smoking has been well described as increasing risk of complications in AP,^13^^-^15 and a suprascarpal dissection intuitively would reduce perfusion to an already ischemic area. We therefore did not offer CGL to any patient who was a smoker. Interestingly, Samra et al^16^ and Neaman et al^10^ reported no increased risk of complications in smokers among those undergoing AP versus lipoabdominoplasty, which indicates that CGL might be possible in smokers, although this would need further study.

Seroma, typically one of the most common complications of AP, was relatively uncommon among our patients. One possible explanation for this is the lack of combined liposuction during our operations, which has been linked to increased seroma formation^10^. In addition, we tend to preserve the layer of deep fat below the level of the inguinal ligament, which has also been suggested as a possible means to lower seroma rate during AP.^16^^,^^17^

We only had 1 patient who requested revision of her AP for a contour irregularity, which compares favorably with revisional rates of up to 36% reported in the literature.^9^^-^13^,^^16^^,^^18^ We do feel that our addition of PBL, with a concomitant reduction in a dog-ear deformity, and CGL, with its favorable contouring of the upper midline groove, result in fewer requests for revision, although this is difficult to measure objectively.

In conclusion, CGL is a safe and efficient technique to achieve an aesthetically pleasing upper midline abdominal contour, with complication rates comparing favorably with those reported in the literature. In addition, for patients who may not be able to afford the additional costs that liposuction adds to standard AP, CGL is a viable alternative in achieving an aesthetically pleasing upper abdominal contour in this subset of patients.
